# History of chronic pain and opioid use is associated with cognitive decline and mild cognitive impairment

**DOI:** 10.1017/S1355617725101057

**Published:** 2025-05

**Authors:** Tyler R. Bell, Jeremy A. Elman, Daniel E. Gustavson, Michael J. Lyons, Christine Fennema-Notestine, McKenna E. Williams, Matthew S. Panizzon, Rahul C. Pearce, Chandra A. Reynolds, Mark Sanderson-Cimino, Rosemary Toomey, Amy Jak, Carol E. Franz, William S. Kremen

**Affiliations:** 1 Department of Psychiatry, University of California, San Diego, La Jolla, CA, USA; 2 Center for Behavior Genetics of Aging, University of California, San Diego, La Jolla, CA, USA; 3 Institute for Behavioral Genetics and Department of Psychology and Neuroscience, University of Colorado Boulder, Boulder, CA, USA; 4 Department of Psychological and Brain Sciences, Boston University, Boston, MA, USA; 5 Memory and Aging Center, Weill Institute for Neurosciences, San Francisco, CA, USA

**Keywords:** Chronic pain, pain interference, cognitive decline, mild cognitive impairment, memory, executive function

## Abstract

**Background:**

The impact of chronic pain and opioid use on cognitive decline and mild cognitive impairment (MCI) is unclear. We investigated these associations in early older adulthood, considering different definitions of chronic pain.

**Methods:**

Men in the Vietnam Era Twin Study of Aging (VETSA; *n* = 1,042) underwent cognitive testing and medical history interviews at average ages 56, 62, and 68. Chronic pain was defined using pain intensity and interference ratings from the SF-36 over 2 or 3 waves (categorized as mild versus moderate-to-severe). Opioid use was determined by self-reported medication use. Amnestic and non-amnestic MCI were assessed using the Jak-Bondi approach. Mixed models and Cox proportional hazards models were used to assess associations of pain and opioid use with cognitive decline and risk for MCI.

**Results:**

Moderate-to-severe, but not mild, chronic pain intensity (*β* = −.10) and interference (*β* = −.23) were associated with greater declines in executive function. Moderate-to-severe chronic pain intensity (*HR* = 1.75) and interference (*HR* = 3.31) were associated with a higher risk of non-amnestic MCI. Opioid use was associated with a faster decline in verbal fluency (*β* = −.18) and a higher risk of amnestic MCI (*HR* = 1.99). There were no significant interactions between chronic pain and opioid use on cognitive decline or MCI risk (all *p*-values > .05).

**Discussion:**

Moderate-to-severe chronic pain intensity and interference related to executive function decline and greater risk of non-amnestic MCI; while opioid use related to verbal fluency decline and greater risk of amnestic MCI. Lowering chronic pain severity while reducing opioid exposure may help clinicians mitigate later cognitive decline and dementia risk.

**Research questions or topics**

- This study examines whether chronic pain intensity and interference, as well as opioid medication use, are associated with cognitive decline and the risk of mild cognitive impairment (MCI), a precursor stage of dementia, in older adults.

**Main findings**

- Moderate-to-severe chronic pain intensity and interference predicted greater declines in executive function and significantly increased the risk of non-memory (non-amnestic) MCI. Opioid medication use independently contributed to declines in verbal fluency and nearly doubled the risk of memory-related (amnestic) MCI.

**Study contributions**

- These results highlight chronic pain and opioid use as distinct factors contributing to cognitive impairment in aging adults. Clinically, the findings underscore the importance of separately evaluating pain severity and opioid medication history to potentially prevent or delay cognitive decline and dementia.

## Background

Chronic pain affects approximately 20% of older adults and is associated with increased dementia risk (Kumaradev et al., 2021; Whitlock et al., 2017) and poorer cognitive performance. Potential mechanisms underlying this relationship include cognitive distraction (Eccleston & Crombez, 1999) and neurodegenerative changes (Apkarian et al., 2009). Despite these known associations, it remains unclear when cognitive impairment linked to chronic pain first manifests. Additionally, most studies have not considered the independent cognitive impact of opioid use, which is common among chronic pain sufferers (Levine et al., 2023; Warner et al., 2022). Prior research has mainly linked chronic pain to episodic memory decline (Veronese et al., 2018; Whitlock et al., 2017), with less attention given to other cognitive domains such as executive function and verbal fluency. Moreover, the relationship between chronic pain and the risk of developing mild cognitive impairment (MCI), specifically amnestic and non-amnestic subtypes, remains understudied.

Chronic pain can be characterized by both intensity – the subjective experience of unpleasant sensation lasting over six months – and interference – the disruption of daily activities due to pain, also lasting over six months (Shega et al., 2010). Both pain intensity and interference have been associated with increased Alzheimer’s disease (AD) and dementia risk (Ezzati et al., 2019; Ikram et al., 2019), yet their impact on MCI risk warrants further investigation.

Opioid use in older adults is linked to increased cognitive impairment and dementia, primarily documented in clinical populations. Epidemiological studies indicate that chronic opioid use confers a greater dementia risk compared to non-users (Gao et al., 2024), along with persistent deficits in working memory and executive functioning (Schiltenwolf et al., 2014). Clinical research further reveals structural brain changes associated with opioids, including reduced gray matter volume in the prefrontal cortex and hippocampus, potentially due to neuroinflammation (Seney et al., 2021; Upadhyay et al., 2010). However, cognitive effects of opioid use in community-dwelling older adults may differ, highlighting the importance of opioid use as a potential confounding factor in chronic pain research (Zajacova et al., 2023).

We investigated how chronic pain intensity and interference relate to domain-specific cognitive decline and incident amnestic and non-amnestic MCI, accounting for opioid use history. Given that cognitive decline is influenced by multiple factors related to chronic pain and opioid use, analyses adjusted for age, physical morbidities, and depressive symptoms, with sensitivity analyses further adjusting for anticholinergic medication use and genetic risk for Alzheimer’s disease (family history, APOE ε4). Based on existing literature (Bell et al., 2018; Whitlock et al., 2017), we hypothesized that chronic pain would predict cognitive decline – particularly in episodic memory and executive function – and increased MCI risk. We explored whether this would be significant for moderate-to-severe versus mild levels of chronic pain intensity and interference. Given evidence linking opioid use to dementia risk (Gao et al., 2024), we also hypothesized that opioid use would primarily relate to declines in executive function and processing speed, as well as increase overall MCI risk.

## Methods

### Participants

Participants were in the Vietnam Era Twin Study of Aging (VETSA) project, an ongoing longitudinal study of adult men beginning in midlife. They were members of the Vietnam Era Twin Registry, a national registry of male adult twins who served at some time during the Vietnam era (1965–1975) (Goldberg et al., 2002; Henderson et al., 1990). All Registry members were invited to participate in the Harvard Drug Study (Tsuang et al., 2001), for which ascertainment was not based on any diagnostic or substance use criteria, and VETSA participants were randomly recruited from the Harvard Drug Study sample. Most of the VETSA sample (∼80%) did not experience combat exposure and are comparable to American men in their age cohort on education, health, and lifestyle factors (Schoeneborn & Heyman, 2009). More details about the VETSA project can be found elsewhere (Kremen et al., 2013; Kremen et al., 2019; Kremen et al., 2006). All procedures were approved by the Institutional Review Boards at the respective study sites and in accordance with Helsinki Declaration.

At Wave 1, there were 1280 individuals with available MCI diagnostic and pain data. For these analyses, we excluded individuals who had MCI at Wave 1 (*n* = 136), had a history of confounding neurological conditions (i.e., stroke, MRI-based cerebral abnormality, multiple sclerosis, HIV/AIDS, seizure disorder, schizophrenia, brain cancer, severe drug or alcohol dependency, *n* = 94), or with missing data on predictors of interest (*n* = 8). This left a final sample of 1,042 participants who were cognitively unimpaired at Wave 1, 82% of whom completed Wave 2 (*n* = 852) and 70% of whom completed Wave 3 (*n* = 729) assessments. Note that some participants only completed wave 1 (*n* = 177) and therefore did not have sufficient data (i.e., more than one timepoint) to classify chronic pain or incident MCI status. However, they were still able to provide information about the sample for descriptive purposes, especially regarding covariates. Average age at each Wave was 55.88 (*SD* = 2.43, range = 51.10 to 6.69), 61.55 (*SD* = 2.40, range = 56.45 to 66.07), and 67.37 (*SD* = 2.56, range = 61.37 to 73.25) years. Thus, the average interval between Wave 1 and Wave 3 was 11.49 years.

### Measures

#### Chronic pain history

Pain was assessed using the SF-36 Quality of Life questionnaire version 1 (Ware & Sherbourne, 1992). This scale includes two items about pain. First, participants are asked about average pain intensity, “How much pain severity have you had during the past 4 weeks?”. Participants answer on a 6-point Likert-type scale selecting “None” (1), “Very Mild” (2), “Mild” (3), “Moderate” (4), “Severe” (5), “Very severe” (6). Second, the pain interference question asked “How much did pain interfere with your normal work (including both work outside the home and housework?” Participants respond from “None at all” (1), “A little bit” (2), “Moderately” (3), “Quite a bit” (4), “Extremely” (5).

Research indicates the SF-36 pain items demonstrate high internal consistency (*α* range: .88–.91) and strong correlations with comprehensive pain scales like the Brief Pain Inventory and McGill Pain Questionnaire (*r* range: .76–.79), supporting their construct validity (Gandek et al., 2004; Jensen et al., 1999; Ware & Sherbourne, 1992). These items are also widely utilized in large-scale epidemiological studies, underscoring their suitability for population-level chronic pain assessment (Breivik et al., 2006; Gandek et al., 2004). The pain interference item specifically aligns well with functional impairment measures, reflecting robust pain-related disability (*r* range: .73–.84; Dworkin et al., 2005). Furthermore, high test-retest reliability (ICC: .85–.90) supports their stable assessment capability over time (McHorney et al., 1993). Given these strong psychometric properties, the SF-36 pain items provide a valid and practical method for chronic pain characterization in this study.

We created two longitudinal pain phenotypes: pain intensity and pain interference. To be classified as “chronic,” participants had to report pain at two or three study waves, representing persistent (all waves) or recurring (waves 1 and 3) pain. This definition aligns with the International Association for the Study of Pain’s criteria for chronic pain (pain lasting ≥3 months; Treede et al., 2019). Mild chronic pain intensity was categorized as responses of “Very Mild” to “Mild,” and moderate-to-severe chronic pain intensity as “Moderate” to “Very Severe,” at multiple waves. These thresholds match commonly accepted clinical ratings used on the Visual Analogue Scale and Numeric Rating Scale (NRS; Boonstra et al., 2014; Chow et al., 2016; Von Korff et al., 2020). Similarly, mild chronic pain interference included responses of “A little bit,” whereas moderate-to-severe interference comprised responses from “Moderately” to “Extremely.” The latter aligns with the CDC National Pain Strategy’s definition of “high-impact chronic pain,” characterized by significant disruption of daily activities (Dahlhamer, 2018).

#### Cognitive performance

Cognitive performance was measured using factor scores of tests within the domains of executive function, episodic memory, processing speed, verbal fluency, and visuospatial ability. The approach to create the cognitive factor scores has been fully detailed and validated across multiple papers using this dataset (Gustavson et al., 2018; Gustavson et al., 2019; Sanderson-Cimino et al., 2019). Just like the cognitive domains they represent, these factor scores are related but distinct (inter-correlations of .37 to .54). Cognitive factor scores were theory-driven and then confirmed by SEM modeling as detailed in the supplemental methods. Factor scores were standardized to the sample mean and standard deviation at Wave 1 and were corrected for practice effects (see Supplemental Material). Higher scores indicate better cognitive abilities. Therefore, a value of 1 indicates someone is performing 1 *SD* above the average performance of the sample at baseline and one unit change represents that a person had a 1 *SD* change in the cognitive ability in units of baseline standard deviations.

#### Young-adult cognitive ability

Data were also collected on general cognitive ability, measured with the Armed Forces Qualifying Test (AFQT) at average age 20 (Lyons et al., 2017; Lyons et al., 2009; Uhlaner & Bolanovich, 1952). This was used as a key covariate in models for cognitive decline to adjust models for young adult level of overall cognitive ability

#### Classification of MCI

MCI at wave 2 and 3 was diagnosed using the Jak-Bondi approach with the 18 neuropsychological tests covering 6 cognitive abilities (Bondi et al., 2014; Jak et al., 2009). There is not complete overlap with the tests in the aforementioned cognitive abilities. For purposes of MCI diagnosis, some measures were averaged into composites to reduce the imbalance in number of tests per ability. This procedure results in 2 to 4 test measures per ability. These tests cover episodic memory (3 tests), executive function (4 tests), attention/working memory (3 tests), verbal/language (2 tests), visuospatial (3 tests), and processing speed (2 tests). The impairment criterion was scoring>1.5 SDs below publisher-provided age-adjusted normative means on 2 or more tasks within a cognitive ability. This threshold is stricter than the more commonly used threshold of >1 SD as we have shown this provides a more reasonable prevalence in our relatively young, community-dwelling sample (Granholm et al., 2017), and have shown this phenotype to be related to AD genetic risk and AD-related brain structure (Logue et al., 2019; Williams et al., 2021).

Prior to applying publisher provided age-based norms, raw test scores were adjusted in two ways. First, they were adjusted for practice effects using the mean differences in scores of returnees and attrition replacements included in other VETSA studies. This methodology is explained in our previous work that showed that accounting for practice effects captured more individuals with incident MCI at follow-up in our sample (Elman et al., 2018) and was more strongly associated with AD biomarkers in the Alzheimer’s Disease Neuroimaging Initiative (Sanderson-Cimino et al., 2022). Secondly, we adjusted for early adulthood general cognitive ability (measured at average age 20) to ensure that scores reflected a decline in performance rather than just longstanding low ability. If there was impairment in episodic memory, MCI was classified as amnestic. Impairment in a cognitive ability other than episodic memory was classified as non-amnestic.

#### Opioid use history

At each wave, participants were asked during an in-person medical interview to name the medications they were prescribed and using. Generally, self-reported medication has shown good agreement with prescription data (Hafferty et al., 2018). Although we do not have more detailed information, yes or no self-reported opioid use can be useful for examination of the effects of opioid “exposure” in clinical care and general population studies (Cron et al., 2017; Warner et al., 2022). We assessed the influence of opioid use history, coded as 1 = “ever having used an opioid medication” versus 0 = “not ever using an opioid medication”. Participants were not asked to report on dosage.

#### Covariates

At each wave, covariates included age, depressive symptoms and medical morbidity. Age was selected as a covariate to account for age-typical cognitive decline. We also adjusted for depressive symptoms because depressive symptoms and physical morbidities are higher in people reporting pain and have also been associated with cognitive decline (John et al., 2019; Sharpe et al., 2017; Wei et al., 2020). Current depressive symptoms were indexed using the 20-item Center of Epidemiological Studies depression scale (CESD-20) (Radloff, 1977). Physical morbidities was measured as the summed index of the following reported conditions (yes/no responses) based on the Charlson Comorbidity index (Charlson et al., 1994): diabetes, emphysema, asthma, cancer, osteoarthritis, rheumatoid arthritis, stroke, heart attack, heart failure, heart surgery, angina, hypertension, peripheral vascular disease, cirrhosis, and AIDS (note some of these are not present due to exclusion criteria described above).

For sensitivity analyses, we included additional covariates. First, we examined the associations when adjusting for exposure to anticholinergic medications, another major class of medication with potential impacts on cognition in addition to opioids. This was done by identifying medications at each wave that had any level of anticholinergic activity based on previous research (Chew et al., 2008). This led to a covariate coded as 1 = “ever having used an anticholinergic medication” versus 0 = “not ever having used an anticholinergic medication.”

We also adjusted for elevated genetic risk for AD by adjusting for family history of dementia and presence of the APOE ε4 allele. Family history was determined by asking the participant about whether their mother or father had a history of AD (yes or no). If both twins reported yes, this was coded 1 and if there was disagreement or both reported no, this was coded 0. Because self-report is likely not specific to AD, this is henceforth referred to as family history of dementia. APOE genotype was determined from blood samples using established methods (Emi et al., 1988; Hixson & Vernier, 1990), APOE genotypes (ε2/ε2, ε2/ε3, ε3/ε3, ε3/ε4, and ε4/ε4) were independently determined twice by laboratory personnel at the VA Puget Sound Healthcare System. For analyses, an APOE-ε4 load measure was created that weights ε4 alleles on the genetic risk of AD, determined from a large genome-wide association study (Leonenko et al., 2021), while accounting for reduced risk due to the presence of ε2 alleles (equation: APOE-ε4 load = −.47*number of e2 alleles + 1.12*number of ε4 alleles).

Furthermore, we additionally examined findings when excluding people who developed dementia. This was measured during the medical history interview during the third wave of the study. Participants were asked if a doctor or physician had ever diagnosed them with dementia. Participants were coded as having dementia if they reported ever having dementia

### Statistical analysis

We used SPSS v.29 (IBM Corp, 2023) to calculate descriptive statistics for continuous (means and standard deviations) and ordinal/categorical (frequencies and percentages) variables at each wave reported in Table 1. SPSS function *genlinmixed* was also used to run logistic linear mixed models assessing associations of demographic variables at wave 3 with having a history of chronic pain phenotypes, as follows: Log(Probability of Chronic Pain History)j ∼ β0j +β1(Age)j + β2(Race)j + β3(General Cognitive Ability at Age 20)j + β4(Depressive Symptoms [CESD-20])j + β5(Physical Morbiditiess)j + β6(Opioid Use History)j + ej, where j = observations nested within twin pairs. A random intercept was included for twin pair to account for correlated outcomes.

**Table 1.** Descriptives of sample at each wave

		Wave 1 *n* = 1,042	Wave 2 *n* = 852	Wave 3 *n* = 729
Age (years)	*M* (*SD*)	55.88 (2.43)	61.55 (2.40)	67.37 (2.56)
Education (years)	*M* (*SD*)	13.98 (2.16)	14.01 (2.16)	14.11 (2.16)
Non-hispanic white	*n* (%)	93.2% (971)	93.0% (792)	93.3% (680)
Income ($10k)	*M* (*SD*)	6.24 (2.95)	5.98 (3.05)	6.03 (3.10)
Family history of dementia	*n* (%)	13.3% (139)	16.1% (137)	18.9% (138)
APOE e4+	*n* (%)	25.8% (269)	31.7% (270)	34.3% (250)
Memory^a^	*M* (*SD*)	.06 (.87)	−.16 (.97)	−.38 (1.02)
Executive function^a^	*M* (*SD*)	.11 (.95)	−.34 (.91)	−.63 (.92)
Speed^a^	*M* (*SD*)	.04 (.90)	−.53 (.95)	−.89 (1.01)
Fluency^a^	*M* (*SD*)	.04 (.97)	−.10 (.97)	−.21 (.99)
Visuospatial ability^a^	*M* (*SD*)	−.10 (.96)	−.24 (.98)	−.49 (.93)
MCI	*n* (%)	–	9.0% (77)	1.2% (74)
Amnestic MCI	*n* (%)	–	4.3% (37)	5.9% (43)
Non-amnestic MCI	*n* (%)	–	4.7% (40)	4.3% (31)
Incident MCI Incident amnestic MCI Incident non-amnestic MCI	*n* (%)		9.0% (77) 4.3% (37) 4.7% (40)	7.4% (54) 3.4% (25) 4.0% (29)
Pain intensity >Mild >Moderate	*n* (%)	35.3% (367) 16.9% (176)	52.3% (446) 28.2% (240)	84.1% (613) 46.9% (342)
Pain interference >Mild >Moderate	*n* (%)	11.8% (123) 4.8% (50)	2.1% (171) 6.1% (52)	36.6% (267) 12.3% (90)
Chronic pain intensity >Mild >Moderate	*n* (%)	–	22.2% (189) 19.5% (166)	24.6% (172) 19.5% (142)
Chronic pain interference >A little bit >Moderately	*n* (%)		9.7% (83) 3.1% (26)	1.2% (75) 2.9% (21)
Physical morbidities^b^ 0 1 2+	*n* (%)	4.3% (420) 33.5% (349) 26.2% (273)	23.9% (204) 31.0% (264) 4.0% (341)	18.0% (131) 28.7% (209) 52.3% (381)
Depressive symptoms (CESD-20)	*M* (*SD*)	7.54 (7.23)	6.57 (7.22)	6.33 (7.02)
Current smoking	*n* (%)	21.9% (228)	1.2% (10)	12.1% (88)
Alcohol consumption^c^ 0 (none) 1 (1–28 drinks) 2 (>28 drinks)	*n* (%)	31.0% (323) 55.2% (575) 13.8% (144)	37.8% (322) 49.5% (422) 12.7% (108)	37.0% (270) 51.2% (373) 11.8% (86)
Opioid medications ≥1	*n* (%)	5.2% (54)	8.9% (76)	5.9% (43)
Anti-cholinergic use ≥1	*n* (%)	13.9% (145)	19.7% (168)	24.6% (179)

*Notes:* AD = Alzheimer’s dementia, CESD-20 = Centers for Epidemiological Studies – Depression Scale 20 items, MCI = mild cognitive impairment.

^a^These represent cognitive factor scores standardized to the mean and standard deviation of the full VETSA sample at baseline.

^b^Physical morbidities is the sum of a subset of major health conditions listed on the Charlson index (diabetes, emphysema, asthma, cancer, osteoarthritis, rheumatoid arthritis, stroke, heart attack, heart failure, heart surgery, angina, hypertension, peripheral vascular disease, cirrhosis, and AIDS).

^c^Alcohol consumption refers to drinks in the last 2 weeks. For our main analyses, we coded people who had any history of chronic pain phenotypes at waves 2 or 3 and any history of opioid use at waves 1, 2, or 3.

Our next analytical goal was to assess whether cognitive change related to a history of chronic pain and opioid use over the three study waves. This was done by using a linear mixed model via SPSS function *genlinmixed*. This model was set up as a multi-level model where Level 1 was as follows: Y(Cognitive Function)ij ∼ β0 + β1(Age centered at 56 _Time-Varying_)ij + β2(Depressive symptoms [CESD-20] centered at mean across waves _Time-Varying_)ij + β3(Physical Morbidities [Physical Morbidities] _Time-Varying_)ij + β4(Chronic Pain History*Age centered at 56 _Time-Varying_)ij + β5(Opioid Use History*Age _Time-Varying_)ij + eij. β4 and β5 can be interpreted as the effect of chronic pain history and opioid history on decline for each additional year of aging, respectively. Level 2 was set up as follows: β0 ∼ γ00i + γ1(Race)ij + γ2(General Cognitive Ability at age 20 [AFQT] centered at the mean )ij + γ3(Chronic Pain History)ij + γ4(Opioid Use History)ij + uij, *i* = observations nested within participants, *j* = participants nested within twin pairs. This model included a random intercept to account for different Wave 1 levels of cognitive function (γ00i). Separate models were run for the four chronic pain phenotypes (mild chronic pain intensity; moderate-to-severe chronic pain intensity; mild chronic pain interference; moderate-to-severe chronic pain interference). In models looking a moderate-to-severe chronic pain intensity and interference, the comparison group consisted of participants with none or mild pain. For models looking at mild chronic pain intensity and interference, the comparison group consisted of participants without chronic pain. Note, to preserve sample size, these comparison groups included individuals who reported pain at one time point but not at another, rather than exclusively those who were pain-free throughout the study. These people would be considered not having chronic pain and inclusion preserved sample size. Sensitivity analyses assessed the interaction of chronic pain phenotype and opioid use [+ β6(Chronic Pain History * Opioid Use History) + β7(Chronic Pain History *Opioid Use History *Age _Time-Varying_)] – as well as additional covariate adjustment for the self-reported anticholinergic medication use, self-reported family history of dementia, and *APOE* ε4 load. We also ran the original model while excluding people who reporting having developed dementia. Effect sizes are provided using standardized betas (βs; small effect ∼ .10; medium ∼ .30; large ∼ .50) (Cohen, 1988).

Next, we examined the association of a history of chronic pain phenotypes and opioid use with rate of progression to MCI. For this analysis, we fitted cox proportional hazards models via SPSS function *coxreg* to predict the age-at-event: either age at MCI diagnosis or age at last follow-up for those who did not progress to MCI during the follow-up period. The primary predictors were history of chronic pain phenotype, history of opioid use, and covariate values at the wave of MCI conversion or the last follow-up for people who did not convert to MCI. Probability(Age at event) ∼ β0 + β1(Age) + β2(Race) + β3(Depressive Symptoms [CESD-20]) + β4(Physical Morbidities) + β5(Chronic Pain History) + β6(History of Opioid Use) + β7(Twin Pair ID) + ej. Twin pair ID was entered as a covariate to account for correlated outcomes among twin pairs. Separate models were run for amnestic and non-amnestic MCI. Sensitivity analyses assessed the interaction of chronic pain phenotype and opioid use [+ β8(Chronic Pain History* Opioid Use History)]. In addition, we additionally assessed association when adjusted for anticholinergic medication use, family history of dementia, and *APOE* ε4 load. We also ran the original model while excluding people who reporting having developed dementia. Results were interpreted as the cumulative hazard ratio (*HR*) of incident MCI in people reporting the corresponding chronic pain phenotype compared to those without. Statistical significance was determined with *p* < .05 and a 95% confidence interval (CI) not including an *HR* of 1. These can be interpreted in terms of effect sizes (small effect ∼ .80 or 1.20; moderate effect ∼ .67 or 1.50; large effect ∼ .50 or 2.00) (Chen et al., 2010).

Note, we did not apply multiple comparison corrections as Type II error is a concern in cases of low prevalence outcomes such as MCI (Dumas-Mallet et al., 2017). Furthermore, the study examines two conceptual variables – chronic pain and opioid use – with different chronic pain variables reflecting threshold distinctions rather than independent tests. Additionally, statistical comparisons were conducted across distinct outcomes, minimizing concerns of familywise error inflation (Perneger, 1998). Given concerns that overly stringent corrections may obscure meaningful associations, statistical perspectives recommend a cautious approach rather than automatic adjustment (Bender & Lange, 2001; Saville, 1990).

## Results

### Sample descriptives

Full sample descriptives are provided in Table 1. At wave 1, pain intensity was rated at mild (35.3%) and moderate-to-severe ranges (16.9%), as was pain interference (mild: 11.8%; moderate-to-severe: 4.8%). Chronic pain intensity was rated at mild (22.2% to 24.6%) and moderate-to-severe (19.5%) ranges at wave 2 and 3. Chronic pain interference was less common but present for mild (9.7% to 12.8%) and moderate-to-severe (2.9% to 3.1%) ranges. Rates of incident MCI at wave 2 and 3 were 9.0% (*n* = 77) and 1.2% (*n* = 74): 4.3% (*n* = 37) and 5.9% (*n* = 53) for amnestic MCI; and 4.7% (*n* = 40) and 4.0% (*n* = 29) for non-amnestic MCI. All groups with mild or moderate-to-severe chronic pain intensity or interference were more likely than people without pain to report greater depressive symptoms (*ORs* range from 1.07 to 1.08, all *p*-values were < .01) or to report taking an opioid medication (*ORs* range from 4.3 to 11.9, all *p*-values were < .01). Those with mild or moderate-to-severe chronic pain intensity or interference did not differ from their counterparts on race/ethnicity, alcohol consumption, or current smoking status (all *p*-values were > .05). However, those with mild or moderate-to-severe chronic pain intensity were more likely to be younger than their counterparts (*ORs* range from .88 to .89, all *p*-values were < .05). Those with mild chronic pain intensity or mild chronic pain interference were also more likely to have lower young adult general cognitive ability (*ORs* range from .81 to .86, all *p*-values were < .05).

### Cognitive function

**Figure 1.**
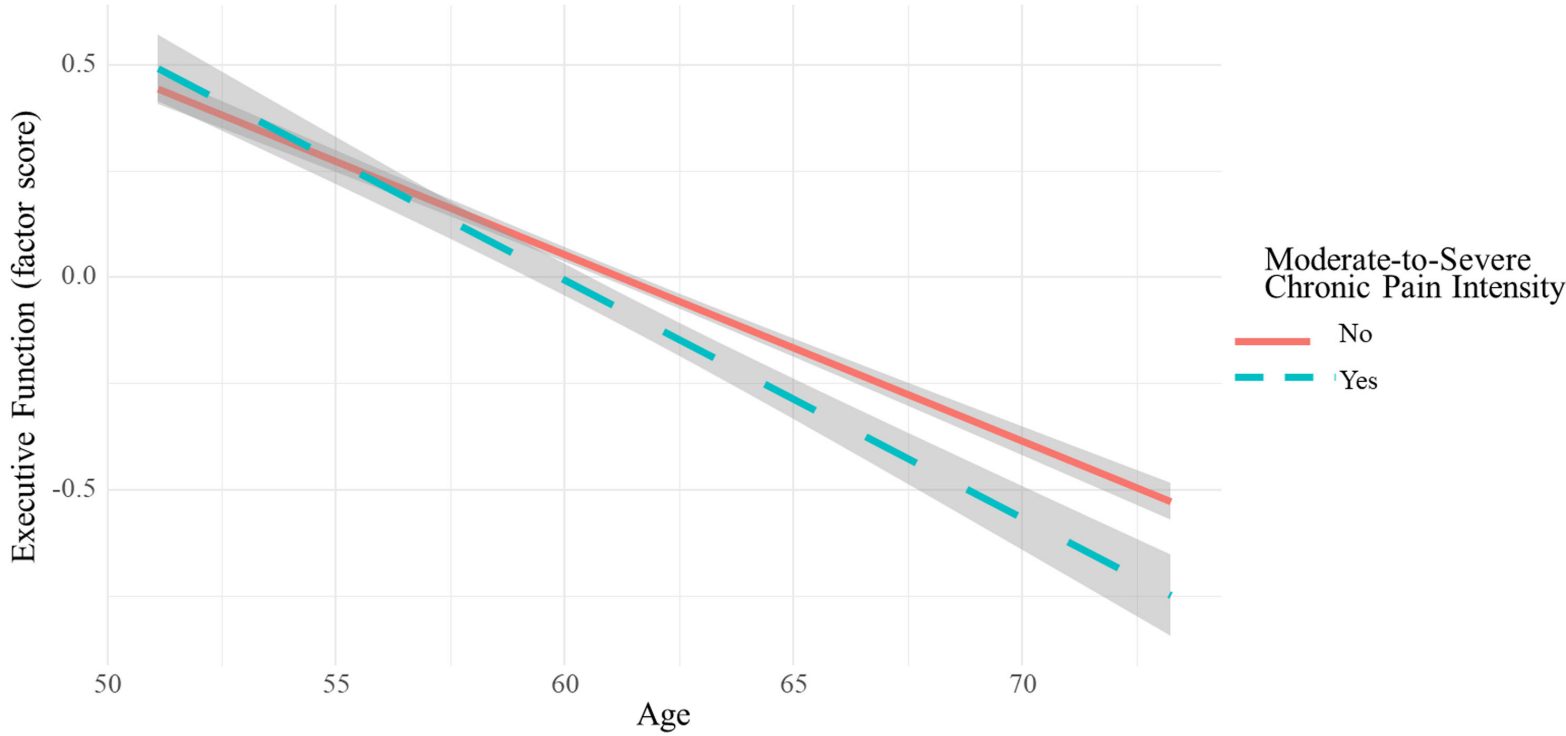
Change in executive function in people with and without moderate-to-severe chronic pain intensity. *Note.* Model shows change in the factor score of executive function estimated by age at follow-up for people with and without a history moderate-to-severe chronic pain intensity (Panel A) and interference (Panel B). Negative slopes indicate cognitive decline. Moderate-to-severe chronic pain intensity is defined as pain intensity from “Moderate” to “Very severe” for 2 or more waves. Moderate-to-severe chronic pain interference is defined as pain interference from “Moderately” to “Extremely” for 2 or more waves.

In the first set of models, we examined how a history of chronic pain intensity and chronic pain interference were associated with cognitive function at the final assessment wave (see Table 2). A history of moderate-to-severe chronic pain intensity was related to worse executive function (*β* = −.10, 95%CI: −.20, −.001, *p* = .047). Likewise, a history of moderate-to-severe chronic pain interference was related to worse executive function (*β* = −.23, −.44 to −.02, *p* = .029). Executive function results are illustrated in Figure 1. A history of mild chronic pain intensity or mild chronic pain interference was not related to any cognitive domain (all *p*-values were > .05). Opioid use history was associated with worse verbal fluency (*β* = −.18, 95%CI: −.30, −.07, *p* = .002).

**Table 2.** Associations of chronic pain history with cognitive change

	Cognitive change (Chronic pain history* age _Time-Varying_)		
Variable	*β*	95%CI	*p*
*Executive function*			
Chronic pain intensity			
Mild	−.02	−.11, .07	.679
Moderate-to-severe	−.10	−.20, −.001	.047
Chronic pain interference			
Mild	−.05	−.17, .07	.444
Moderate-to-severe	.23	−.44, −.02	.029
Chronic pain interference			
Mild	−.04	−.15, .07	.487
Moderate-to-severe	−.24	−.43, −.05	.012
*Episodic memory*			
Chronic pain intensity			
Mild	.05	−1.30, 1.41	.938
Moderate-to-severe	−1.58	−3.17, .02	.053
Chronic pain interference			
Mild	.20	−1.76, 2.16	.843
Moderate-to-severe	−.87	−4.28, 2.53	.615
*Processing speed*			
Chronic pain intensity			
Mild	.51	.99, 2.02	.507
Moderate-to-severe	−.84	−2.61, .93	.353
Chronic pain interference			
Mild	.84	−1.34, 3.01	.449
Moderate-to-severe	−.05	−3.81, 3.72	.981
*Verbal fluency*			
Chronic pain intensity			
Mild	−.02	−.11, .07	.722
Moderate-to-severe	.001	−.10, .10	.980
Chronic pain interference			
Mild	.04	−.08, .17	.522
Moderate-to-severe	−.11	−.32, .11	.331
*Visuospatial ability*			
Chronic pain intensity			
Mild	−.03	−.04, .10	.402
Moderate-to-severe	−.02	−.10, .06	.596
Chronic pain interference			
Mild	−.02	−.11, .08	.749
Moderate-to-severe	−.09	−.27, .08	.283

*Note:* Linear mixed models control for time-varying age, time-varying depressive symptoms using the Center for Epidemiological Studies-Depression Scale 20 items (CESD-20), race (non-Hispanic White versus Other), general cognitive ability at age 20, current smoking status (yes or no), alcohol use (none, mild, moderate-to-severe), and number of physical morbidities based on a subset of conditions from the Charlson Index. CESD-20, current smoking status, alcohol use, and number of medical morbidities were time-varying across waves. Separate models were conducted for each chronic pain intensity and chronic pain interference variables under each cognitive domain.

*β’s represent the interaction term in the linear mixed model of chronic pain with time-varying age which assesses change in the cognitive domain due to chronic pain status. These are standardized estimates and represent effect sizes.

### Mild cognitive impairment

#### Amnestic MCI

As shown in Table 3, a history of chronic pain intensity or chronic pain interference of any severity was not related to greater risk of incident amnestic MCI (all *p*-values were > .05). However, a history of opioid use was related to an increased likelihood of progression to amnestic MCI (*HR* = 1.99, 95%CI: 1.05, 3.77, *p* = .036). Among with a history of opioid use, 16.9% developed amnestic MCI compared to 7.7% not taking an opioid medication.

**Table 3.** Associations between chronic pain and risk of MCI subtypes

	Amnestic MCI			Non-amnestic MCI		
Variable	*HR*	95%CI	*p*	*HR*	95%CI	*p*
Chronic pain intensity						
Mild	1.05	.58, 1.87	.883	1.08	.59, 2.00	.801
Moderate-to-severe	1.46	.58, 3.80	.170	1.75	1.004, 3.06	.049
Chronic pain interference						
Mild	.97	.48, 1.98	.934	1.13	.48, 2.64	.780
Moderate-to-severe	.77	.19, 3.23	.724	3.31	1.44, 7.62	.005
Opioid use	1.99	1.05, 3.77	.036	1.70	.84 to 3.45	.138

*Note:* CI = confidence interval, MCI = mild cognitive impairment. Cox-regression models control for depressive symptoms using the Center for Epidemiological Studies-Depression Scale 20 items (CESD-20), race (non-Hispanic White versus Other), current smoking status (yes or no), alcohol use (none, mild, moderate-to-severe), and number of physical morbidities based on a subset of conditions from the Charlson Index, provided at Wave 3. The measure of MCI was already adjusted for general cognitive ability at age 20 (AFQT).

#### Non-amnestic MCI

**Figure 2.**
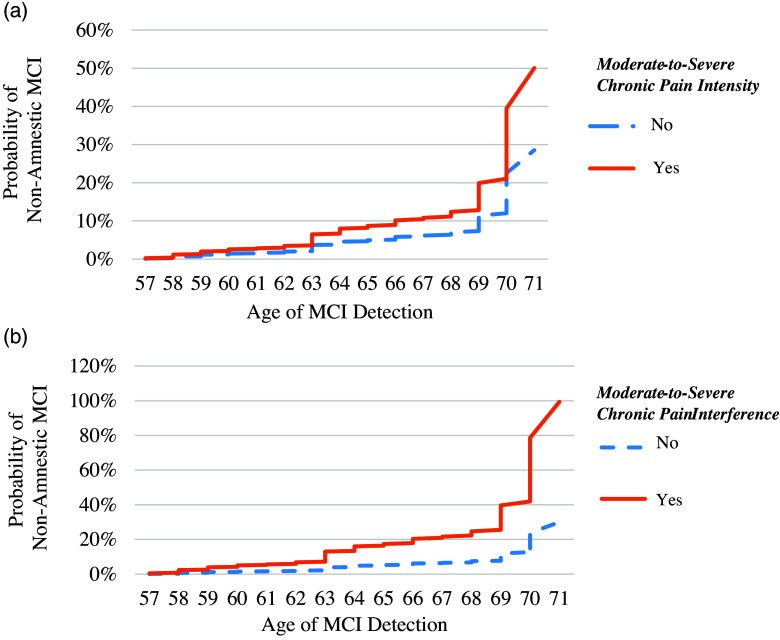
Risk of non-amnestic mild cognitive impairment in people with and without moderate-to-severe chronic pain intensity (Panel A) and moderate-to-severe chronic pain interference (Panel B). *Note.* MCI = mild cognitive impairment. Mild cognitive impairment is defined as performing greater than 1.5 standard deviations below average performance on multiple tests within a cognitive domain. Age of detection is based on the age at the study wave when the participant first was detected to have MCI. Moderate-to-severe chronic pain intensity is defined as pain intensity from “Moderate” to “Very severe” for 2 or more waves. Moderate-to-severe chronic pain interference is defined as pain interference from “Moderately” to “Extremely” for 2 or more waves.

As shown in Table 3, a history of moderate-to-severe chronic pain intensity was related to increased risk of progression to non-amnestic MCI (*HR* = 1.75, 95%CI: 1.004, 3.06, *p* = .049). Of those with moderate-to-severe chronic pain intensity history, 11.6% developed non-amnestic MCI compared to 7.8% with mild or no chronic pain intensity. Moderate-to-severe chronic pain interference was associated with a three-fold increased risk of progression to non-amnestic MCI (*HR* = 3.31, 95%CI: 1.44, 7.62, *p* = .005). Of those with a history of moderate-to-severe chronic pain interference, 24.1% developed non-amnestic MCI compared to 8.0% with mild or no chronic pain interference. Differences in cumulative hazards are illustrated in Figure 2, panel A and B. A history of mild chronic pain intensity or mild chronic pain interference was unrelated to the risk of progression to non-amnestic MCI (all *p*-values were > .05). A history of opioid use was also unrelated to incident non-amnestic MCI risk (*p* = .138).

### Sensitivity analyses

Sensitivity analyses revealed that there no significant interactions between chronic pain phenotypes with opioid use on risk of non-amnestic MCI, amnestic MCI, or cognitive change (all *p*-values < .05). Furthermore, adjustment for the self-reported anti-cholinergic medication use, family history of dementia, and the *APOE* ε4 allele revealed no differences in the observed pattern of associations. Furthermore, 6 individuals (1%) reported having been given a diagnosis of dementia by Wave 3. Excluding these individuals did not alter the pattern of associations.

## Discussion

Chronic pain has been linked to increased dementia risk. We examined how chronic pain intensity and interference relate to cognitive decline and mild cognitive impairment (MCI) risk in cognitively healthy, community-dwelling men initially in their 50s, also considering opioid use as a potential confounder and independent risk factor. As hypothesized, chronic pain intensity and inference – but only of moderate-to-severe levels – predicted declines specifically in executive function over an 11.5-year follow-up and increased non-amnestic MCI risk, with moderate-to-severe chronic pain interference tripling this risk. Contrary to our initial expectations, chronic pain was not strongly linked to episodic memory decline or amnestic MCI. Interestingly, opioid use was associated with greater verbal fluency decline and doubled the risk of amnestic MCI, suggesting distinct cognitive consequences for chronic pain versus opioid use.

Our findings on moderate-to-severe chronic pain specifically affecting executive function and non-amnestic MCI align with prior studies (Berryman et al., 2014; Grisart & Plaghki, 1999; Veronese et al., 2018), though some previous research noted broader cognitive impacts (Legrain et al., 2009; van der Leeuw et al., 2016). The absence of episodic memory impairment might be due to the relatively younger age of our sample, given that executive deficits often precede memory impairments (Carlson et al., 2009; Schiltenwolf et al., 2014). Neuroimaging studies suggest cognitive resources are redirected toward pain processing at higher pain intensities (Davis et al., 1997). Thus, managing moderate-to-severe pain could significantly preserve cognitive health and lower MCI risk, though continued follow-up may reveal later-emerging memory effects.

Although beyond the scope of our study, two mechanisms may underlie our findings on chronic pain. First, chronic pain may interfere with cognitive resources due to its attention-demanding nature; the cognitive-affective model suggests pain processing is prioritized for survival, reducing resources for other cognitive tasks (Eccleston & Crombez, 1999). Second, higher chronic pain intensity could induce neurodegeneration, especially in regions such as the prefrontal cortex critical for executive function (Apkarian et al., 2009; Lorenz et al., 2003), consistent with our observations linking pain intensity to non-amnestic MCI risk.

Opioid use independently increased MCI risk, but unexpectedly this risk was specific to amnestic rather than non-amnestic MCI. Opioid use correlated with declines in verbal fluency, potentially signaling future episodic memory impairment as previously suggested (Gustavson et al., 2020). Lack of observed effects on executive function might reflect lower opioid exposure levels in our community-based sample compared to previous clinical studies (Schiltenwolf et al., 2014), and possibly nonlinear cognitive effects of high-dose opioids (Dublin et al., 2015). Several mechanisms might explain opioid-associated cognitive impairment. Opioids may induce cognitive decline through neuroinflammation, increasing hippocampal inflammatory markers (IL-1β, IL-6, TNFα) observed in animal studies (Muscat et al., 2021). Chronic opioid use also increases amyloid-beta (Aβ) production, a hallmark of Alzheimer’s disease, by elevating amyloid precursor protein and BACE1 expression, further enhancing neuroinflammation (Sil et al., 2021). Additionally, long-term opioid use reduces gray and white matter integrity (Upadhyay et al., 2010; Wollman et al., 2017) and impairs hippocampal neurogenesis essential for cognitive maintenance (Zhang et al., 2016).

No significant interaction was found between chronic pain and opioid use, suggesting they independently contribute to cognitive impairment rather than having synergistic effects. Clinically, these findings emphasize assessing chronic pain and opioid use separately to identify distinct cognitive decline profiles. Future research should explore how opioid dosage, treatment duration, specific pain management strategies, and subgroup differences (e.g., individuals with opioid use disorder) might influence these relationships. Importantly, these findings should not discourage appropriate opioid use for pain management among older adults, a group often undertreated for pain.

Our study has several limitations that also provide avenues for future research. The sample was exclusively male and predominantly white, limiting generalizability. Although causality between chronic pain and MCI cannot be confirmed, previous studies suggest causal pathways involving inflammation or amyloid and tau pathology (Cao et al., 2019; Moore et al., 2012). Measurement limitations included using general pain questions that restrict evaluating specific pain subtypes. Furthermore, opioid use was self-reported and did not include information on dosage and regimen. This measure may not capture the nuances of opioid use, such as dosage, duration, and context of use, which can be critical for understanding the full impact of opioid exposure (Carrell et al., 2020). However, our study does show that general exposure to opioids elevates risk of amnestic MCI. Also, there was an unequal number of tests for different cognitive abilities, which may influence prevalence of non-amnestic versus amnestic MCI. However, we attempted to minimize the number of tests using composites to reduce this possibility.

Analytical limitations include potential influences from unmeasured medications and health conditions, though we adjusted for cardiovascular risks and employed strict exclusion criteria. We also did not assess mediators, such as sleep disorders, mobility decline, and health behaviors, which may explain cognitive decline due to chronic pain – but these remain important areas for future research that could identify valuable intervention targets (Cao et al., 2024; Halloway et al., 2024; Mathias et al., 2018; Rose et al., 2014; Suh et al., 2018). Another limitation is that our observed effect sizes between chronic pain and cognitive decline (βs < .20) were modest; but this is similar to other dementia risk factors like APOE ε4 (Marioni et al., 2016; Wisdom et al., 2011). Nonetheless, the Effect sizes for MCI associations were moderate to large (*HR*s: 1.75–1.99).

Our study addressed key methodological limitations in prior research, such as cross-sectional designs, limited cognitive assessments, small samples, and short follow-up periods (Attal et al., 2014; Bergh et al., 2003; James & Ferguson, 2020; Shega et al., 2010; Veronese et al., 2018; Westerbotn et al., 2008). We utilized a large sample, comprehensive cognitive battery, multiple confounder adjustments, and extensive follow-up exceeding suggested minimum durations (de Aguiar et al., 2020).

In conclusion, chronic pain and opioid use appear to be distinct and significant risk factors for cognitive decline and MCI. Moderate-to-severe chronic pain predominantly increased risk for non-amnestic MCI, whereas opioid use increased amnestic MCI risk. Further research is needed to clarify underlying biological mechanisms. Continued follow-up of this relatively young cohort may reveal additional cognitive risks at older ages, informing strategies for pain management to mitigate cognitive decline and dementia risk.

## Supporting information

Bell et al. supplementary material Bell et al. supplementary material

## Supplementary material

For supplementary material accompanying this paper visit https://doi.org/10.1017/S1355617725101057.
