# Supplementary material for: History of chronic pain and opioid use is associated with cognitive decline and mild cognitive impairment
Source: J Int Neuropsychol Soc. 2025 Jul 14;31(4):307–17. doi: 10.1017/S1355617725101057 (PMC12404075; doi:10.1017/S1355617725101057)
Supplement: Bell et al. supplementary material [file S1355617725101057sup001.docx]

## Supplemental Methods

## *Cognitive Performance*

Cognitive performance was measured using factor scores of tests within the domains of executive function, episodic memory, processing speed, verbal fluency, and visuospatial ability. The approach to create the cognitive factor scores has been fully detailed and validated across multiple papers using this dataset (Gustavson, Panizzon, Franz, et al., 2018; Gustavson et al., 2019; Sanderson-Cimino et al., 2019). Just like the cognitive domains they represent, these factor scores are related but distinct (inter-correlations of .37 to .54). Cognitive factor scores were theory-driven and then confirmed by SEM modeling as follows:

**Episodic memory:** The episodic memory factor score is based on the combined number of correctly recalled words on the short and long delay conditions and the total number of words recalled across the five learning trials (i.e., the sum of all correct responses across learning trials 1 through 5) of the California Verbal Learning Test-II (CVLT-II) (Delis et al., 2001b) and the combined number of correctly recalled story details on the immediate and delayed portions of the Wechsler Memory Scale-III Logical Memory test and the WMS-III Visual Reproductions test (Wechsler, 1997). A SEM model of the larger VETSA sample has shown good overall fit for the latent variable model used to derive the episodic memory factor score (CFI = .98, TLI = .97, RMSEA = .04) (Bell et al., 2023). More details on these measures and factor score creation are available in prior work (Gustavson et al., 2020; Kremen et al., 2014).

**Executive function:** We derived a factor score of executive function using measures of inhibition (Color-Word trial of the Golden and Freshwater (2002) Stroop test statistically adjusted for Color and Word trials for speed); shifting (reaction time on Condition 4 of the Delis-Kaplan Executive Function System Trail Making task, statistically adjusted for statistically adjusted for Number and Letter Sequencing tasks, Conditions 2 and 3) (Delis et al., 2001), and working memory span (total number of trials completed on the Letter-Number Sequencing and Digit Span tasks from Wechsler Memory Scale (Wechsler, 1997). A SEM model of the larger VETSA sample has shown good overall fit for the latent variable model used to derive the executive function factor score (CFI = .98, TLI = .97, RMSEA = .03) (Gustavson, Panizzon, Elman, Franz, Reynolds, et al., 2018). More details on these measures and factor score creation are available in prior work (Gustavson, Panizzon, Elman, Franz, Reynolds, et al., 2018).

**Verbal fluency:** D-KEFS Letter and Category fluency were used to capture verbal fluency. The factor score was derived from the total number of correctly named words in six Letters and Categories trials. A SEM model of the larger VETSA sample has shown good overall fit for the latent variable model used to derive the verbal fluency factor score (CFI = .99, TLI = .96; RMSEA = .03) (Bell et al., 2023). More details on these measures and factor score creation are available in prior work (Gustavson, Panizzon, Elman, Franz, Beck, et al., 2018).

**Visuospatial ability:** Visuospatial ability was captured using accuracy scores on the Gottschaldt Hidden Figures task (Gottschaldt, 1929) and the WMS-III Visual Reproductions Copy task (Wechsler, 1997). We calculated a SEM model from the larger VETSA sample and found that the latent variable model used to derive the visuospatial factor score had good overall fit (CFI = .97, TLI = .95; RMSEA = .03) ( Bell et al., 2023; Tang et al., 2024).

**Processing Speed:** Processing speed was captured using six processing speed measures from three tests: the Delis-Kaplan Executive Function System Trail-Making Test (D-KEFS) number sequencing and letter sequencing (Delis et al., 2001), Simple Reaction Time (Nesselroade, 2010), and the Stroop word and color conditions (Golden and Freshwater, 2002). More details on these measures and factor score creation are available in prior work (Sanderson-Cimino et al., 2019).

For waves 2 and 3, all individual test raw scores were adjusted for practice effects before factor score calculation. As described in previous work, raw test scores were pre-adjusted for practice effects based on mean differences in performance between returnees and attrition replacement participants recruited beginning at wave 2 (Elman et al., 2018). The adjustments could be applied to all participants although replacement participants were not included in the present analyses because inclusion began with participants in wave 1. Raw practice-adjusted test scores were then weighted based the previous CFAs to create the factor scores. Factor scores were standardized to the sample mean and standard deviation at Wave 1. Higher scores indicate better cognitive abilities. Therefore, a value of 1 indicates someone is performing 1 *SD* above the average performance of the sample at baseline and one unit change represents that a person had a 1 *SD* change in the cognitive ability in units of baseline standard deviations.

## *Classification of MCI*

MCI at wave 2 and 3 was diagnosed using the Jak-Bondi approach with the 18 neuropsychological tests covering 6 cognitive abilities (Bondi et al., 2014; Jak et al., 2009). There is not complete overlap with the tests in the aforementioned cognitive abilities. For purposes of MCI diagnosis, some measures were averaged into composites to reduce the imbalance in number of tests per abiilty. This procedure results in 2 to 4 test measures per ability. These tests cover episodic memory (3 tests: composite of CVLT-II Sum of trials 1-5 and delayed free recall; composite of WMS-III Logical Memories Immediate and Delayed Free Recall; composite of WMS-III Visual Reproduction Immediate and Delayed Free Recall), executive function (4 tests: D-KEFS Trails: Switching, Condition 4; D-KEFS Fluency Category Switching; Stroop Color-Word, interference; Weshler Abbreviated Scale of Intelligence (WASI) Matrix Reasoning), attention/working memory (3 tests; WMS-III Digit Span; WMS-III Letter Number Sequencing; D-KEFS Trails Visual Scanning [Condition 1]), verbal/language (2 tests: D-KEFS Letter Fluency; D-KEFS Category Fluency), visuospatial (3 tests: Gottschaldt Hidden Figures; Mental Rotation; WMS-III Visual Reproduction Copy), and processing speed (2 tests: D-KEFS Trails composite of Number Sequencing and Letter Sequencing, also known as Conditions 2 and 3; composite of Stroop Word and Stroop Color conditions). The impairment criterion was scoring >1.5 SDs below publisher-provided age-adjusted normative means on 2 or more tasks within a cognitive ability. This threshold is stricter than the more commonly used threshold of >1 SD as we have shown this provides a more reasonable prevalence in our relatively young, community-dwelling sample (Granholm et al., 2017), and have shown this phenotype to be related to AD genetic risk and AD-related brain structure (Logue et al., 2019; Williams et al., 2021).

Prior to applying publisher provided age-based norms, raw test scores were adjusted in two ways. First, they were adjusted for practice effects using the mean differences in scores of returnees and attrition replacements included in other VETSA studies. This methodology is explained in our previous work that showed that accounting for practice effects captured more individuals with incident MCI at follow-up in our sample (Elman et al., 2018) and was more strongly associated with AD biomarkers in the Alzheimer’s Disease Neuroimaging Initiative (Sanderson‐Cimino et al., 2022). Secondly, we adjusted for early adulthood general cognitive ability (measured at average age 20) to ensure that scores reflected a decline in performance rather than just longstanding low ability. If there was impairment in episodic memory, MCI was classified as amnestic. Impairment in a cognitive ability other than episodic memory was classified as non-amnestic.

References

Bell, T.R., Elman, J.A., Beck, A., Fennema-Notestine, C., Gustavson, D.E., Hagler, D.J., Jack, A.J., Lyons, M.J., Puckett, O.K., Toomey, R., Franz, C.E., & Kremen, W.S. (2023). Rostral-middle locus coeruleus integrity and subjective cognitive decline in early old age. *J Int Neuropsychol Soc,* *29*(8), 763-774.

Bondi, M.W., Edmonds, E.C., Jak, A.J., Clark, L.R., Delano-Wood, L., McDonald, C.R., Nation, D.A., Libon, D.J., Au, R., Galasko, D., & Salmon, D.P. (2014). Neuropsychological criteria for mild cognitive impairment improves diagnostic precision, biomarker associations, and progression rates. *J Alzheimers Dis,* *42*(1), 275-289.

Delis, D.C., Kaplan, E., & Kramer, J.H. (2001b). *Delis-Kaplan Executive Function System (D-KEFS)*. San Antonio, TX: Psychological Corporation.

Golden, C. J., & Freshwater, S. M. (2002). *Stroop Color and Word test: A manual for clinical and experimental uses*. Stoelting.

Gottschaldt, K. (1929). Über den Einfluss der Erfahrung auf die Wahrnehmung von Figuren. II. Vergleichende Untersuchungen über die Wirkung figuraler Einprägung und den Einfluss spezifischer Geschehensverläufe auf die Auffassung optischer Komplexe. *Psychologische Forschung*.

Granholm, E.L., Panizzon, M.S., Elman, J.A., Jak, A.J., Hauger, R.L., Bondi, M.W., Lyons, M.J., Franz, C.E., & Kremen, W.S. (2017). Pupillary responses as a biomarker of early risk for Alzheimer’s disease. *Journal of Alzheimer’s Disease,* *56*(4), 1419-1428.

Gustavson, D.E., Elman, J.A., Sanderson-Cimino, M., Franz, C.E., Panizzon, M.S., Jak, A.J., Reynolds, C.A., Neale, M.C., Lyons, M.J., & Kremen, W.S. (2020). Extensive memory testing improves prediction of progression to MCI in late middle age. *Alzheimers Dement (Amst),* *12*(1), e12004.

Gustavson, D.E., Panizzon, M.S., Elman, J.A., Franz, C.E., Beck, A., Reynolds, C.A., Jacobson, K.C., Xian, H., Toomey, R., Lyons, M.J., & Kremen, W.S. (2018). Genetic and environmental influences on verbal fluency in middle age: A longitudinal twin study. *Behav Genet,* *48*(5), 361-373.

Gustavson, D.E., Panizzon, M.S., Franz, C.E., Reynolds, C.A., Corley, R.P., Hewitt, J.K., Lyons, M.J., Kremen, W.S., & Friedman, N.P. (2019). Integrating verbal fluency with executive functions: Evidence from twin studies in adolescence and middle age. *J Exp Psychol Gen,* *148*(12), 2104-2119.

Jak, A.J., Bondi, M.W., Delano-Wood, L., Wierenga, C., Corey-Bloom, J., Salmon, D.P., & Delis, D.C. (2009). Quantification of five neuropsychological approaches to defining mild cognitive impairment. *American Journal of Geriatric Psychiatry,* *17*, 368-375.

Kremen, W.S., Panizzon, M.S., Franz, C.E., Spoon, K.M., Vuoksimaa, E., Jacobson, K.C., Vasilopoulos, T., Xian, H., McCaffery, J.M., Rana, B.K., Toomey, R., McKenzie, R., & Lyons, M.J. (2014). Genetic complexity of episodic memory: A twin approach to studies of aging. *Psychol Aging,* *29*(2), 404-417.

Logue, M.W., Panizzon, M.S., Elman, J.A., Gillespie, N.A., Hatton, S.N., Gustavson, D.E., Andreassen, O.A., Dale, A.M., Franz, C.E., & Lyons, M.J. (2019). Use of an Alzheimer’s disease polygenic risk score to identify mild cognitive impairment in adults in their 50s. *Molecular Psychiatry,* *24*(3), 421-430.

Sanderson-Cimino, M., Panizzon, M.S., Elman, J.A., Gustavson, D.E., Franz, C.E., Reynolds, C.A., Toomey, R., Lyons, M.J., & Kremen, W.S. (2019). Genetic and environmental architecture of processing speed across midlife. *Neuropsychology,* *33*(6), 862-871.

Sanderson‐Cimino, M., Elman, J.A., Tu, X.M., Gross, A.L., Panizzon, M.S., Gustavson, D.E., Bondi, M.W., Edmonds, E.C., Eglit, G.M., & Eppig, J.S. (2022). Cognitive practice effects delay diagnosis of MCI: Implications for clinical trials. *Alzheimer's & Dementia: Translational Research & Clinical Interventions,* *8*(1), e12228.

Tang, R., Buchholz, E., Dale, A.M., Rissman, R.A., Fennema-Notestine, C., Gillespie, N.A., Hagler, D.J., Jr., Lyons, M.J., Neale, M.C., Panizzon, M.S., Puckett, O.K., Reynolds, C.A., Franz, C.E., Kremen, W.S., & Elman, J.A. (2024). Associations of plasma neurofilament light chain with cognition and neuroimaging measures in community-dwelling early old age men. *Alzheimers Res Ther,* *16*(1), 90.

Wechsler, D. (1997b). *WAIS-III: Wechsler Adult Intelligence Scale*. San Antonio, TX: Psychological Corporation

Williams, M.E., Elman, J.A., McEvoy, L.K., Andreassen, O.A., Dale, A.M., Eglit, G.M., Eyler, L.T., Fennema-Notestine, C., Franz, C.E., & Gillespie, N.A. (2021). 12-year prediction of mild cognitive impairment aided by Alzheimer’s brain signatures at mean age 56. *Brain communications,* *3*(3), fcab167.
